# Isolation and identification of a novel *Bacillus velezensis* strain JIN4 and its potential for biocontrol of kiwifruit bacterial canker caused by *Pseudomonas syringae* pv. *actinidiae*

**DOI:** 10.3389/fpls.2024.1513438

**Published:** 2024-12-23

**Authors:** Xin Zhao, Yang Zhai, Lin Wei, Fei Xia, Yuanru Yang, Yongjian Yi, Hongying Wang, Caisheng Qiu, Feng Wang, Liangbin Zeng

**Affiliations:** ^1^ Institute of Bast Fiber Crops, Chinese Academy of Agricultural Sciences, Changsha, China; ^2^ Institute of Plant Protection, Hunan Academy of Agricultural Sciences, Changsha, China; ^3^ College of Computer and Mathematics, Central South University of Forestry and Technology, Changsha, Hunan, China; ^4^ Guizhou Academy of Tobacco Science, Guizhou, China

**Keywords:** antibacterial activity, *Bacillus velezensis*, biocontrol agent, kiwifruit bacterial canker, whole genome sequencing

## Abstract

Bacterial canker is a devastating disease in kiwifruit production, primarily caused by *Pseudomonas syringae* pv. *actinidiae*. In this study, a strain of *Bacillus velezensis* named JIN4, isolated from a kiwifruit branch, showed antagonistic activity. The *B*. *velezensis* JIN4 strain was identified based on its morphological, physiological and biochemical characteristics, 16S rDNA, and *gyrA* sequences. Furthermore, the complete genome of the strain was elucidated, revealing the presence of three genes that confer resistance to antibiotics, including tetracycline, lincomycin, and streptomycin. Additionally, a cluster of nine secondary metabolite synthesis genes was identified. In the laboratory, the JIN4 strain exhibited nitrogen (N) and phosphorus (P) production, demonstrating plant growth-promoting and broad-spectrum inhibitory activities against pathogenic fungi and bacteria. In the field, the JIN4 strain demonstrated effective colonization of kiwifruit, increased antioxidant enzyme activity, induced resistance in kiwifruit plants, and achieved a control efficiency of 60.22% against kiwifruit bacterial canker. These results indicate that *B*. *velezensis* JIN4 is a potential biocontrol agent against kiwifruit bacterial canker.

## 1 Introduction

Kiwifruit bacterial canker is one of the most serious diseases in kiwifruit production worldwide (Sawada and Fujikawa, 2019). This disease is caused by *Pseudomonas syringae* pv. *actinidiae* (*Psa*). Since its discovery, the disease has spread rapidly across the globe and is difficult to prevent and control (Vanneste, 2017). Apart from antibiotics and copper agents, few bactericides are available to control *Psa*. The frequent use of antibiotics and copper agents has accelerated the development of resistance in this pathogen and caused environmental and animal-related issues. Control of kiwifruit bacterial canker primarily relies on growing resistant kiwifruit varieties, using standard pruning management practices, spraying chemicals before the onset of wound flow, and timely tree spraying during the growing and dormant periods (Vanneste et al., 2011; Mauri et al., 2016). Such control measures can reduce field incidence by maintaining the number of *Psa* pathogens at a low level. Still, this technique is highly dependent on the management skills of the grower. Biological control bacteria have been found to have high potential in the control of cankers. A strain of *B*. *velezensis* WL-23 isolated from kiwifruit branches in Guizhou Province, China, was effective against bacterial and fungal pathogens of kiwifruit in Guizhou (Wang et al., 2023).

Biological control represents an environmentally friendly plant protection strategy that relies on the use of biocontrol bacteria to inhibit or control plant pathogens (Huang et al., 2017). Genome sequencing and enzyme activity assays are pivotal technologies for elucidating the mechanism of action of biocontrol bacteria and optimizing their deployment. Genome sequencing can provide information on the genetic background of biocontrol fungi, thus facilitating the identification of genes and metabolic pathways related to disease resistance (Zhou et al., 2021). For example, whole genome sequencing enables the identification of clusters of genes encoding bacteriostatic substances, including surface active agents, fungens, polyenes, catechol-type ferredoxins, and others, in biocontrol bacteria (Eshboev et al., 2024). The mining of these genetic resources provides a theoretical basis for the development of novel biopesticides and a deeper understanding of the bacteriostatic mechanisms of bioprotective bacteria. Concurrently, the associated enzyme activity assay serves as a direct indicator for evaluating the impact of plant resistance elicited by biocontrol bacteria. Modifications in the activities of defensive enzymes, including polyphenol oxidase (PPO), peroxidase (POD), superoxide dismutase (SOD), phenylalanine ammonia-lyase (PAL) and catalase (CAT), can be indicative of the impact of biocontrol fungi on plant disease resistance. The activities of these defence enzymes were enhanced by the treatment of biocontainment fungi, indicating that biocontainment fungi were able to induce stronger disease resistance in plants. This induced resistance not only enhances plant defence against specific pathogens, but may also have a broad-spectrum effect against a wide range of pathogens.

This study isolated a strain of *B*. *velezensis* named JIN4 from the kiwifruit branches in Hunan Province, China. We identified this strain using morphological, physiological, and molecular methods. The control efficacy of JIN4 to kiwifruit bacterial canker disease was evaluated in the laboratory and the fields.

## 2 Materials and methods

### 2.1 Strain isolation

Branches were collected from healthy kiwifruit plants in Changsha City, Hunan Province, China, where kiwifruit bacterial canker is the main disease during the bleeding period in the spring. The collected branches were washed with sterile water, followed by surface sterilization with 75% alcohol for 30 s, and sterile water washing three times. About 1 g of bark from the branch was ground into a slurry under aseptic conditions using an automatic sample grinding instrument (Shanghai Jingxin Industrial Development Co., Ltd., JXFSTPRP 24) by adding 9 mL of water and one gram of quartz sand. The slurry was diluted 10, 100, and 1000 times using sterile distilled water. A total of 100 μL of each dilution was added to lysogeny broth (LB) agar medium (agar 20 g, tryptone 10 g, NaCl 10 g, yeast extract 5 g in 1000 mL of sterile water at neutral pH) and incubated at 25°C for 72 h. Three replicates were conducted for each dilution concentration.

Based on the morphology of the cultures, six species of *Bacillus subtilis* were initially identified. These *Bacillus* isolates from kiwifruit were screened using pure culture (Cho et al., 2011) after morphological identification using a stereoscopic microscope to pick out purified Bacillus. Briefly, 100 μL of C48 pathogen (*Psa*) was spread evenly on a 20 mL plate (90 mm diameter) of King’s B (KB) solid medium (peptone 20 g, agar 15 g, glycerol 10 mL, K_2_HPO_4_ 1.5 g, and MgSO_4_·7H_2_O 1.5 g in 1000 mL sterile water at neutral pH). The bacterial isolate was then inoculated at six symmetrical sites 2.5 cm from the center of the KB solid plate. Plates inoculated only with the pathogen were used as controls. Each treatment was replicated three times. The plates were incubated in the dark at 25°C for 48 h and observed. The strongest antagonistic activity against the C48 pathogen was identified as JIN4 (**Supplementary Table 1**).

### 2.2 Morphological identification

After activation, JIN4 was inoculated onto KB solid medium plates and incubated for 24 h at 30°C to observe the growth of the colonies. A Gram Stain Kit (Morin et al., 2014) and a Spore Stain Kit (Beijing Solarbio Science & Technology Co., Ltd.) were used for routine Gram and budding staining of JIN4. The activated JIN4 was inoculated in a KB liquid medium (without agar; the others are the same as KB solid medium) and incubated at 30°C on a shaker at 160 r/min for 24 h. The culture medium was aspirated at an optical density of 0.5–0.8 and centrifuged. Then, the medium was discarded, and the bacterial sediment was collected at the bottom of the tube with 0.1 mol/L, pH = 7.0 phosphate-buffered saline (PBS), washed twice, where the supernatant was discarded, and a pre-cooled fixative (25% glutaraldehyde 10 mL, 0.2 mol/L PBS 50 mL, add water to 100 mL) was slowly added along the wall of the tube at 4°C and stored at 4°C for 12 h. After this, the fixative was decanted, and the sample was rinsed with 0.1 mol/L, pH = 7.0, phosphate buffer (PB) for 15 min three times. Then, the samples were fixed by soaking them in a 1% osmiridic solution for 2 h. The waste solution was discarded, and the samples were rinsed with PBS buffer for 15 min and rinsed three times. The dehydrated samples were soaked in six ethanol solutions of 30%, 50%, 70%, 80%, 90%, and 95% in descending order for 15 min and then treated with 100% ethanol twice for 20 min. A mixture of ethanol and isoamyl acetate (V/V=1:1) was used to soak the samples for 30 min, and then 100% isoamyl acetate was used to soak and treat the samples for 1 h. The samples were dried at the critical point of CO_2_, plated with gold films, and observed using scanning electron microscopy, which was used for the collection of micrographs (Hick and Preece, 1990; Nakagawa et al., 2000; Saxeń, 2011; Novozhilov et al., 2022).

### 2.3 Molecular identification using 16S rDNA and the *gyrA* gene

Strain JIN4 was further identified by amplifying 16S rDNA sequences using polymerase chain reaction (PCR) with the universal bacterial primers 27F (5’-AGA GTT TGA TCC TGG CTC AG-3’) and 1492R (5’-GGT TAC CTT GTT ACG ACT T-3’) (Zhang et al., 2014; Tang et al., 2020). The PCR amplification system (25 μL) contained 5 μL of DNA template, 12.5 μL of 2 × Es Taq MasterMix (CoWin Biosciences Co., Ltd., Beijing, China), 1 μL of each primer, and 5.5 μL of ddH_2_O. PCR conditions were as follows: 94°C for 2 min, 35 cycles of 94°C for 30 s, 55.4°C for 30 s, 72°C for 30 s, and a final extension at 72°C for 2 min. We also sequenced the *gyrA* gene of the JIN4 strain for molecular identification using the specific primers *gyrA*-F (5′-CAG TCA GGA AAT GCG TAC GTC CTT-3′) and *gyrA*-R (5′-CAA GGT AAT GCT CCA GGC ATT GCT-3′) (Borshchevskaya et al., 2013; Khademi et al., 2021). The PCR system was configured in the same manner as 16S rDNA. The PCR reaction was annealed at 55°C. The other PCR conditions were the same as those of 16S rDNA. The PCR products were purified and sequenced by Beijing Tsingke Biotech Co., Ltd.

The obtained 16S rDNA and *gyrA* sequences were compared with the sequences in the GenBank nucleic acid database of NCBI for BLAST homology analysis (https://blast.ncbi.nlm.nih.gov/Blast.cgi) and compared with the reference sequences using Mega 7.0 software. Next, a phylogenetic tree was constructed using the neighbor-joining method bootstrap 1000 (Kuroha et al., 2009; Akter et al., 2022).

### 2.4 Genome sequencing and annotation

A single colony of JIN4 was inoculated in 50 mL of KB liquid medium and incubated at 28°C for 18 h at 160 r/min, with three replicates. The precipitate was collected by centrifugation at 4000 r/min for 3 min, washed twice with 0.01 mol/L PBS buffer (pH 7.2) to remove the medium, and stored at −80°C. The strains were stored on dry ice and sent to Biomarker Technologies (Beijing, China) for bacterial genome sequencing (Harris and Okoro, 2014).

The JIN4 genome was sequenced using PacBio RSII, a third-generation sequencing platform. PacBio sequencing uses SMRT chips as the sequencing vector and four-color fluorescent labeling of the four bases (dNTPs), and the type of bases entered can be determined based on the wavelengths and peaks of different base-pairing lights. Genome assembly was performed using Hifiasm (Cheng et al., 2021), and gene prediction was performed using Prodigal v2.6.3. Subsequent data analysis for mapping was performed using the Biomarker Cloud platform (https://www.biocloud.net).

Non-coding RNAs are RNA molecules that are transcribed from the genome but are not translated and expressed into proteins, such as mRNAs, ribosomal RNA (rRNA), transfer RNA (tRNA), and other non-coding RNAs, which are referred to as other-ncRNAs in this study. Predictive identification of non-coding RNAs was performed using Infernal v1.1.3 and tRNAscan-SE v2.0 (Nawrocki and Eddy, 2013) in combination with Rfam and the tRNAscan-SE database (Ioanna et al., 2017; Chan and Lowe, 2019). Clustered, regularly interspaced palindromic repeats (CRISPR) is a contiguous region of DNA in the genome that interferes with the replicative action of exogenous DNA and protects bacteria from the invasion of external DNA. We used CRT v1.2 (Bland et al., 2007) for CRISPR genome prediction.

Annotation analysis of the genome based on the gene ontology (GO) database and the Kyoto Encyclopedia of Genes and Genomes (KEGG). Annotation of drug resistance genes based on the Comprehensive Antibiotic Resistance Database (CARD). The most widely used tool for the identification and analysis of biosynthetic gene clusters within bacterial and fungal genomes, antiSMARSH version 6.0.1 database online analysis (https://antismash.secondarymetabolites.org) (Blin et al., 2021), was used to analyze the predicted strains, the main secondary metabolites of JIN4, their gene cluster locations, and the use of the TBtools tool to map Circos to mark secondary metabolite gene cluster locations (Chen et al., 2020).

### 2.5 Physiological measurements and characterization of functional substances

The following common physiological and biochemical characteristics (Tan et al., 2018; Zhu et al., 2020) of JIN4 were determined considering Bergey’s Manual of Determinative Bacteriology (Holt et al., 1994) and The Manual for the Identification of Common Bacterial Systems, including the catalase test, oxidase, starch hydrolysis, methyl red (MR), Voges–Proskauer test (VP), gelatin liquefaction, nitrate reduction, H_2_S production, citrate utilization, lecithinase test, malonate utilization, cellulolytic activity and fermentation utilization of sugars, containing glucose, D-galactose, D-arabinose, D-mannose, D-fructose, D-xylose, respectively (Ma et al., 2002; Wen et al., 2015; Moawia et al., 2016). Some biocontrol bacteria can also promote plant growth and development through the secretion of growth-promoting substances, such as indole-3-acetic acid (IAA) and siderophore, and the use of such growth-promoting bacteria can strengthen seedlings (Bach et al., 2016; Lau et al., 2020; Zhai et al., 2021). JIN4 was characterized for its functional substances and its ability to produce IAA (Maor et al., 2004; Nunes et al., 2023), phosphorus-dissolving ability (Nautiyala, 1999; Wang et al., 2020), and ammonia production capacity (Richard et al., 2018; Liu et al., 2019) was measured.

### 2.6 Antagonistic spectrum

The antagonistic spectrum of JIN4 was obtained by testing the antagonism assay with eight other pathogens (including *Colletotrichum gloeosporioides*, *Fusarium oxyporum f.* sp. *Nelumbicola, Fusarium oxysporium f.* sp. *Cucumerinum, Sclerotinia sclerotiorum, Fusarium oxysporum f.* sp. Lini*, Colletotrichum Linicolum, Phytophthora capsica, Phytophthora nicotianae*) in face-to-face culture on potato dextrose agar (PDA) (potato 200 g, glucose 20 g, agar 15 g, sterile water to 1000 mL, pH neutral) medium (El-Hasan et al., 2018). PDA medium was used to activate these pathogens, 5 mm of the pathogenic cake was inoculated in the center of a 9 cm diameter PDA plate, and the biocontrol fungus JIN4 was spotted 2.5 cm from the center of the plate. The control plate was not inoculated with JIN4; each treatment was replicated three times. The plates were incubated at 28°C for 72 h. The inhibition effect was observed and measured using the cross-patch method.

### 2.7 Pathogens of the kiwifruit bacterial canker

The C48 pathogen (GenBank: MZ336040.1) used in this study was isolated from kiwifruit ulcer samples (Hunan Province, China) in our laboratory and preserved on KB agar medium (Mazumdar et al., 2007) slants at 4°C (Vicente et al., 2004). Before being used in the experiments, this pathogen was grown on KB agar plates at 25°C for two days in the dark.

### 2.8 Inhibition zone test of pathogen of bacterial canker

A standoff culture of C48 was used to assess the inhibition capacity of the bacterium JIN4 in Petri dishes using the crossover method. The pathogenic bacteria JIN4 and C48 were scribed and activated in LB and KB liquid media and incubated at 160 r/min for 36 h at 25°C. A volume of 100 μL of C48 was inoculated and spread on a KB solid medium, and the surface was blown dry. A 6 mm diameter sterile paper pad was dipped in JIN4 and placed at four symmetrical spots. The diameter of the zone of inhibition, centered on the paper pad, was measured after 72 h of incubation at 25°C. The test was repeated in three sets.

### 2.9 Field trial evaluation of JIN4’s effectiveness in controlling kiwifruit diseases

On March 18, 2021, kiwifruit plots planted with hybrid kiwifruit varieties (Yongshun County, Xiangxi Tujia, and Miao Autonomous Prefecture, Hunan) were selected for consistent management levels. All kiwifruit plants were grouped (16 plants per group), and ulcer disease was counted in each group before application. The JIN4 bacteria solution diluted to Optical densit_600_ (OD_600_) = 0.2 and 500 times fine break (3% benziothiazolino, Xian Hytech Agrochemicals Co., Ltd.) was applied to each group of kiwifruit plants in 10 L batches, and the three groups were repeated. The disease incidence scoring of each group was investigated again 14 days after application, and the disease index, disease growth rate, and relative control efficiency were calculated. The kiwifruit bacterial canker field disease incidence scoring criteria are shown in **Table 1**.

**Table 1:** The kiwifruit bacterial canker disease incidence scoring criteria in the field.

Incidence level	Criteria for incidence scoring
0	The plants are healthy and disease free.
1	No spots on the trunk, < 5% incidence on branches and leaves.
2	A few spots on the trunk and a 5%–20% incidence on branches and leaves.
3	Disease spots around the trunk 1/4–1/2, branches and leaves of the disease rate of 20%–50%.
4	Disease spots around the trunk 1/2~3/4, branches and leaves of the disease rate of 50%–80%.
5	Disease spots around the trunk are more than 3/4, and branches and leaves of the disease rate more than 80%.

#### 2.9.1 Equations


$$\begin{array}{c} Disease\;index\\ =\sum^{}\left(\frac{number\;of\;branches\;per\;level\;of\;disease\times \text{disease level}}{number\;of\;branches\;of\;all\;plants\;surveyed\times highest\;level\;of\;disease}\right)\times 100 \end{array}$$

$$\begin{array}{c} Disease\;growth\;rate\left(\%\right)\\ =\left(\frac{disease\;index\;after\;treatment-\text{disease index before treatment}}{disease\;index\;before\;treatment}\right)\times 100 \end{array}$$

$$\begin{array}{c} Control\;efficiency\left(\%\right)\\ =\left(\frac{disease\;growth\;rate\;\left(CK\right)-\text{disease growth rate }\left(\text{Experimental group}\right)}{disease\;growth\;rate\;\left(CK\right)}\right)\times 100 \end{array}$$

### 2.10 Induced kiwifruit resistance

Bacterial suspensions of JIN4 and C48 with OD_600_ = 0.2 were prepared using a KB culture medium. The test was conducted using potted seedlings of Cuiyu kiwifruit with a growth period of two years (3 pots per treatment, four plants per pot), and the pathogen C48 was inoculated 24 h earlier than the biocontrol bacterium JIN4 (experimental design, **Table 2**). Kiwifruit leaves were punctured with the tip of a 1mL sterile syringe prior to inoculation. A clean watering can was used to spray the prepared bacterial suspension spray evenly onto the leaves during inoculation until it began to drip water. Three leaves were taken for treatment 3 and treatment 4 on 1 d, and 3 formed leaves were taken for four groups of treatments on 2 d, 4 d, 6 d, 8 d, and 10 d and stored in a freezer at −80 °C after liquid nitrogen quick-freezing for the determination of PAL, POD, SOD, PPO, CAT, malondialdehyde (MDA), hydrogen peroxide (H_2_O_2_) content.

**Table 2:** Potted kiwifruit treatment design.

Treatment	0 h	24 h
1 (CK)	water	water
2 (C48)	C48	water
3 (C48+JIN4)	C48	JIN4
4 (JIN4)	water	JIN4

The potted kiwifruit treatments were divided into four groups and sprayed with the appropriate bacterial solution at 0 and 24 hours, as shown in the table above.

The kiwifruit leaves were ground into fine powder under liquid nitrogen quick-freezing conditions and weighed into 0.1 g tubes, which were stored at −80°C for later use. 0.1 g of the aliquoted sample was added to 1 mL of 0.01 mol/L PBS buffer (pH = 7.2) and vortexed for 30 s. The supernatant was centrifuged at 4°C, 3500 r/min for 10 min, and the supernatant was used for POD, CAT, SOD, enzyme activity assay, and protein quantification. The sample was centrifuged at 10000 r/min for 10 min at 4°C. Subsequently, the supernatant was collected and used to determine the content of H_2_O_2_ and MDA. PAL and PPO crude enzyme solution extraction uses the extraction solution that comes with the kit.

## 3 Results

### 3.1 Morphological characteristics of strain JIN4

**Figure 1:**
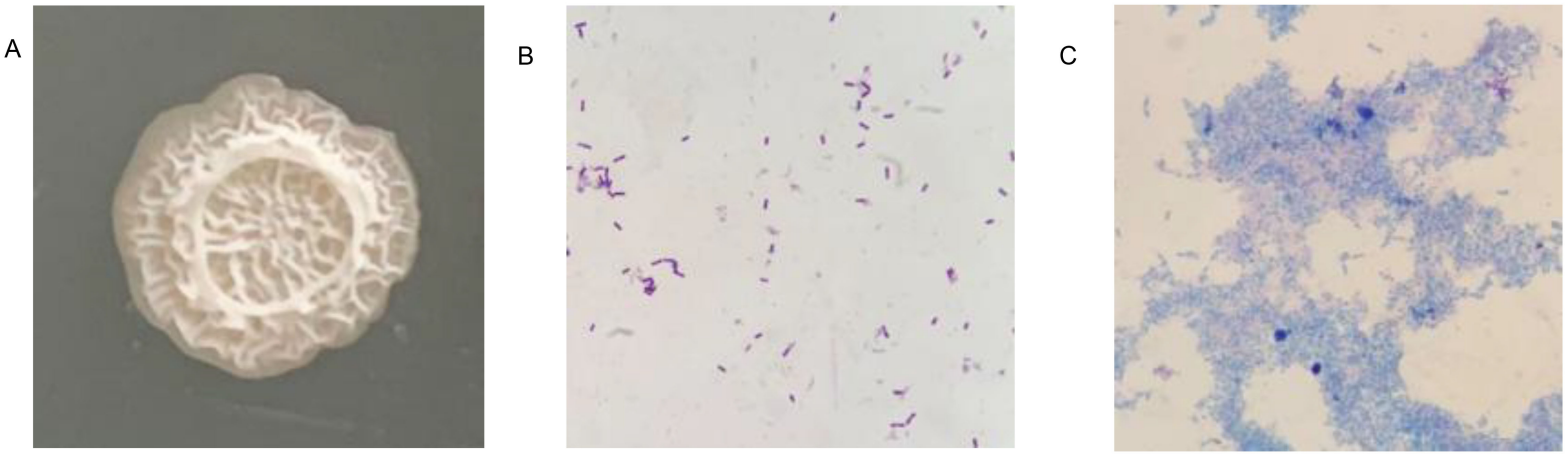
Morphological characteristics of JIN4. **(A)** The growth pattern of JIN4 is irregularly rounded with a white body and a dry, wrinkled, raised crater-like surface. **(B)** JIN4 is a Gram-positive bacterium. **(C)** JIN4 has spores.

**Figure 2:**
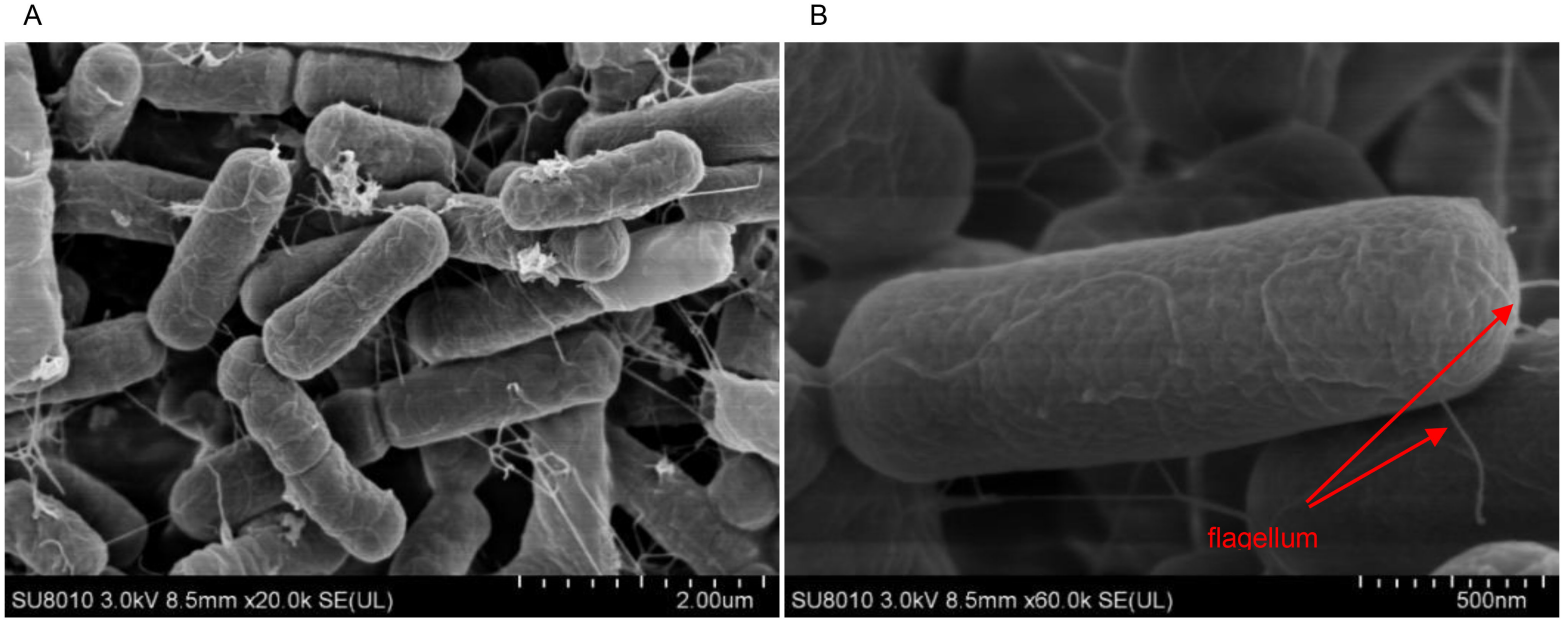
Photography of JIN4 incubated onto KB solid medium plates after 24 h by scanning electron microscope (SEM). **(A)** 20,000 x. **(B)** 60,000 x.

The growth pattern of strain JIN4 was irregularly rounded at the edges, white, and with a dry, wrinkled surface, raised similar to a crater on the KB agar plates (**Figure 1A**). JIN4 for Gram-positive bacterium under microscopic observation (**Figure 1B**). A budding stain test revealed the presence of spore production in JIN4 (**Figure 1C**). Under scanning electron microscopy, JIN4 was found to have long rod-shaped cells with bluntly rounded ends, measuring 0.4–0.8 μm × 1–3 μm (**Figure 2A**), with sparse surrounding flagella and non-expanded sporangium (**Figure 2B**).

### 3.2 Identification of the 16S rDNA sequence and *gyrA* gene sequence of JIN4

**Figure 3:**
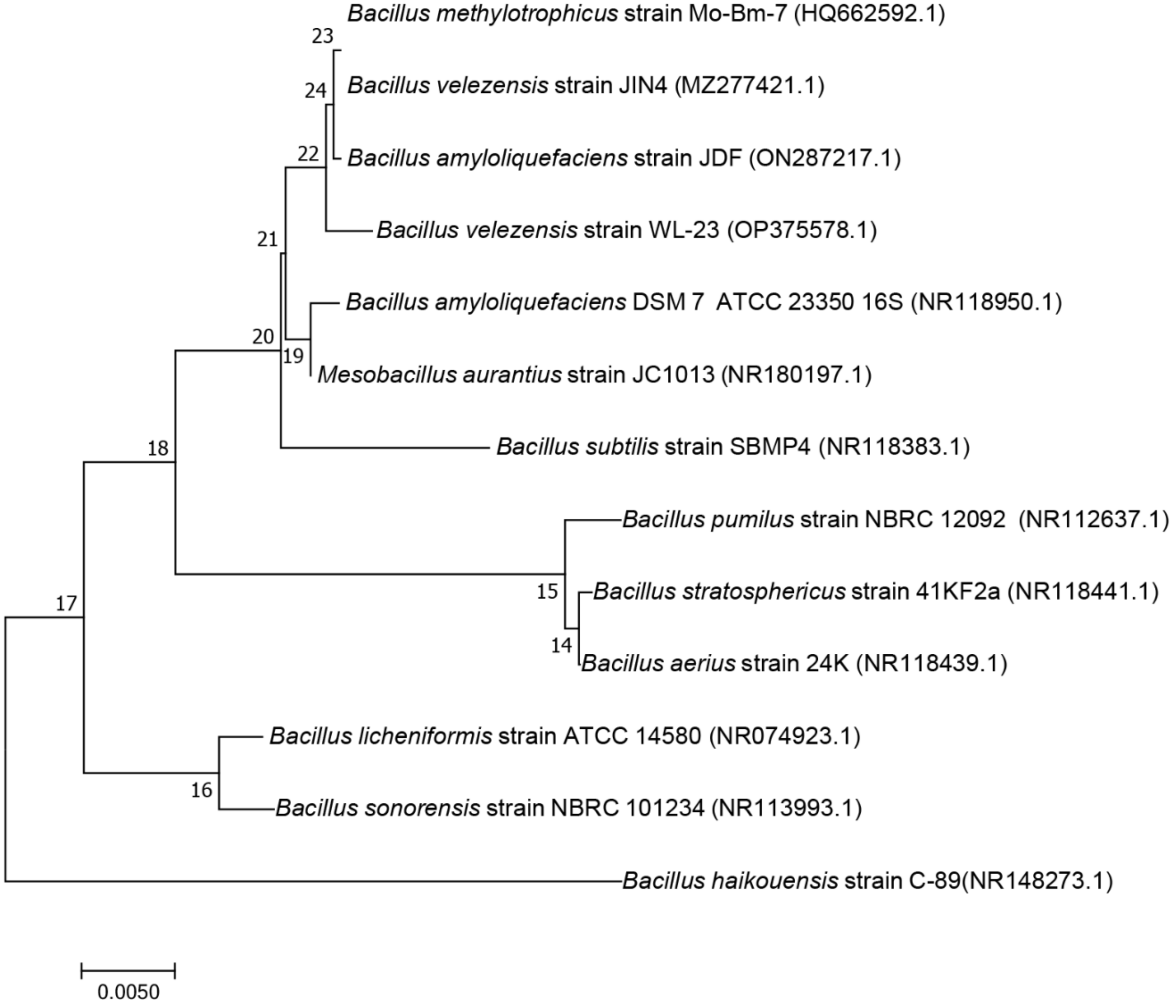
Phylogenetic tree of the antagonist bacterium JIN4 16S rDNA gene sequence.

**Figure 4:**
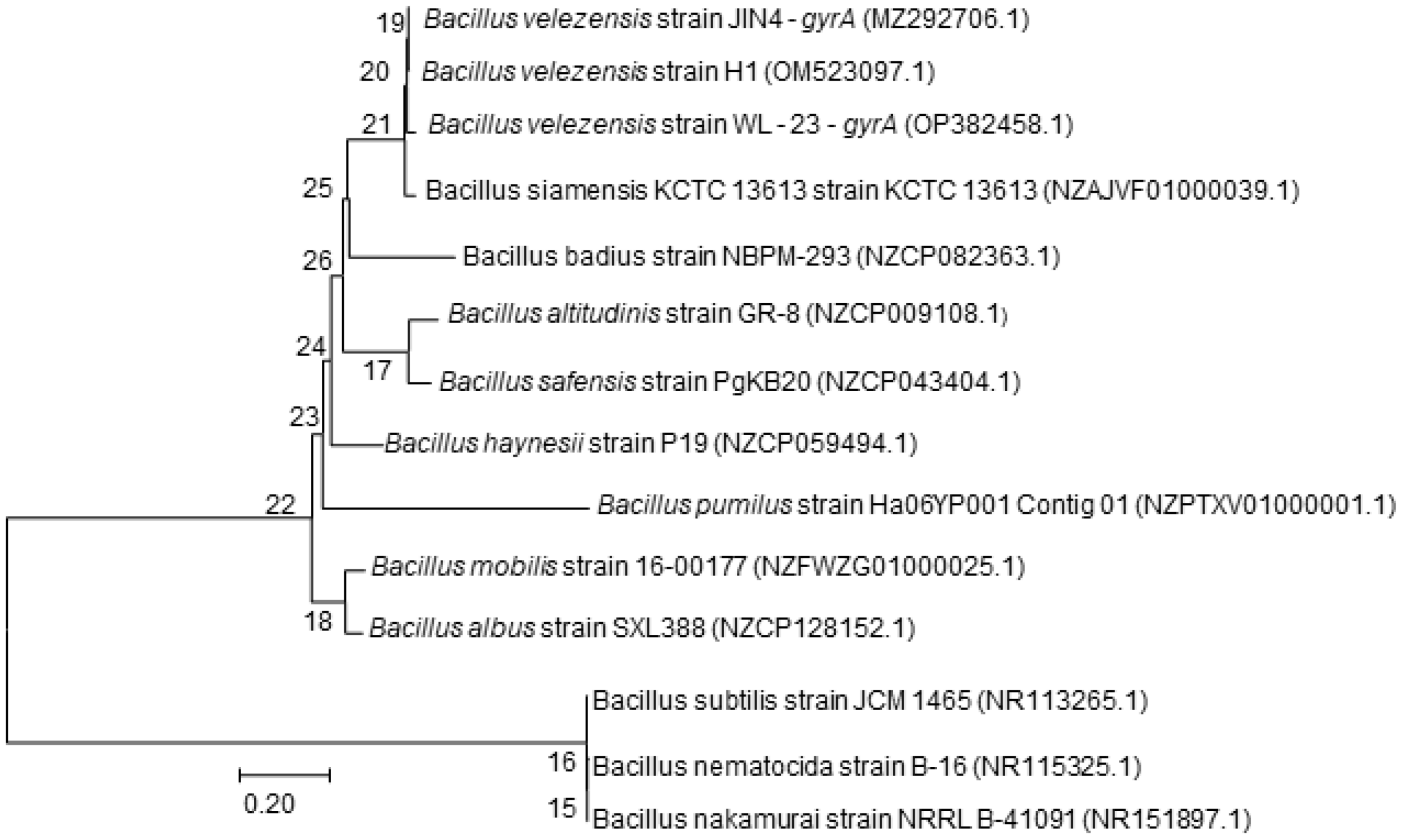
Phylogenetic tree of the antagonist bacterium JIN4 *gyrA* gene sequence.

The 16S rDNA sequence and *gyrA* gene sequence were analyzed using the online BLAST in NCBI GenBank to further identify strain JIN4. The 16S rDNA sequence was found to exhibit 99% identity with the 16S rDNA sequence of *B. velezensis, B. amyloliquefaciens, B. stercoris*, *B. vallismortis*, and *B. amyloliquefaciens*. The phylogenetic tree based on the 16S rDNA sequences indicated that strain JIN4 (MZ277421.1) clustered with *B. subtilis* strain SBMP4 (NR 118383.1) (**Figure 3**) (Serrao et al., 2024). The results from these tests suggested that strain JIN4 belonged to *Bacillus*. The 16S rRNA is highly conserved and may not be able to distinguish between closely related species. Using sequence differences in the *gyrA* gene proved to be a valuable tool for distinguishing closely related *Bacillus* species. The phylogenetic tree based on the *gyrA* sequences indicated that strain JIN4 clustered with *B. velezensis* strain H1 (OM523097.1) (**Figure 4**). The JIN4 finally identified as *B. velezensis*.

### 3.3 Genome features of *B. velezensis* strain JIN4

The whole genome of *B. velezensis* strain JIN4 (GenBank: CP087137.1) has a genome size of 3,904,585 bp, 1 chromosome, and 46.57% of GC content. There are 3734 predicted genes, with an average gene length of 925 bp and a total gene length of 3,454,302 bp, accounting for 88.47% of the total genome length. Repetitive sequences had a predicted length of 4411 bp, representing 0.11% of the genome size. JIN4 was predicted to have nine each of 5S rRNA, 16S rRNA, and 23S rRNA. 5S rRNA contains 116 bp, 16S rRNA contains 1550 bp, and 23S rRNA contains 2930 bp (**Supplementary Table 2**). Predictions on JIN4 are that there are 86 tRNAs with an average length of 77.1 bp. In addition to rRNA and tRNA, many other non-coding RNAs (ncRNAs) such as sRNA, snRNA, and miRNA, were also present in strain JIN4, with a total of 80 predicted, with an average length of 142.5 bp.

The CRISPR analysis of strain JIN4 predicts a total of 12 CRISPR regions, which are key components of the bacterial immune system. Bacteria use these gene clusters to detect and resist attacks from fragmented viruses and destroy their DNA, thereby achieving viral resistance (**Supplementary Table 3**). The CARD annotation predicted resistance for JIN4 and identified the types of antibiotics tolerated (**Supplementary Table 4**), providing an important reference for synergistic antibiotics in biocontrol applications. Three resistance genes were identified in JIN4, showing resistance to the following antibiotics: tetracycline, lincosamide, pleuromutilin, macrolide, oxazolidinone, streptogramin, and phenicol.

Of the 3734 genes predicted for Bacteroides JIN4, 67.65% were annotated based on the GO database (**Supplementary Figure 1**), with 11 branches related to cellular components, 13 for molecular function, and 15 for biological processes. Nine branches contained more than 500 enriched genes, those involved in catalytic activity, metabolic processes, cellular processes, binding, single-organism processes, membranes, membrane parts, cells, and cell parts, comprising 1438, 1238, 1058, 1025, 963, 829, 785, 763, and 749, respectively.

**Figure 5:**
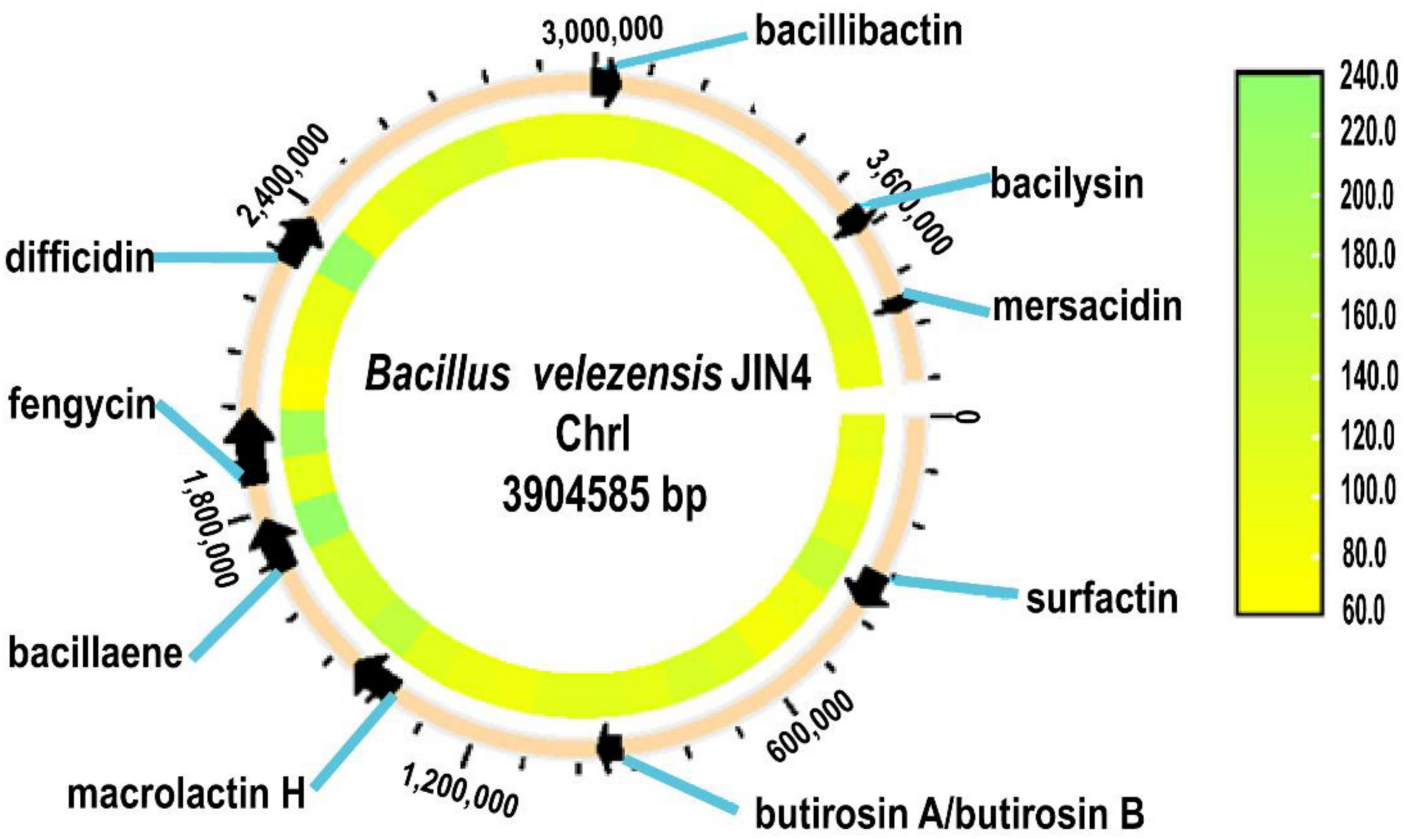
Gene cluster map of secondary metabolites of JIN4.

According to the analysis of this strain in the KEGG database (**Supplementary Figure 2**), 57.87% of the genes were annotated, and the genes were enriched in four metabolic pathways: metabolism, environmental information processing, genetic information processing, and cellular processes. Seven metabolic pathways were enriched with more than or equal to 50 genes, in descending order of ABC transporters, biosynthesis of amino acids, two-component systems, carbon metabolism, quorum sensing, purine metabolism, and ribosomes, which comprised 123, 120, 115, 99, 70, 62, and 56 gene annotation results. The synthesis of active compounds with resistance from secondary metabolites during growth is a disease control mechanism in *B. biotrophicus*. Using antiSMASH to predict the JIN4 genome (**Figure 5**), nine secondary metabolite gene clusters were predicted. These were surfactin, butirosin (A/B), difficidin, bacillaene, fengycin, bacillibactin, bacilysin, macrolactin H, mersacidin, and various peptidoglycan and polyketide compounds.

### 3.4 Physiological characteristics of JIN4

The physiological and biochemical characteristics of strain JIN4 is listed in **Table 3**. Strain JIN4 showed positive reactions in the catalase test, starch hydrolysis, VP, gelatin liquefaction, nitrate reduction, H_2_S production, citrate utilization, lecithin enzyme activity, cellulolytic activity, fermentation utilization tests for glucose, D-mannose, D-fructose, and D-xylose. However, it was negative for the oxidase reaction, MR test, malonate utilization, D-galactose, and D-arabinose utilization. Based on these characteristics, JIN4 was found to be significantly similar to *Bacillus subtilis* (Manju and Shanmugam, 2013; Moawia et al., 2016; Kadiri et al., 2023). Qualitative detection of functional substances in JIN4 showed that strain JIN4 can produce IAA, phosphorus-dissolving, and ammonia production, promoting plant growth and development.

**Table 3:** Statistics of physiological measurements and characterization of functional substances of strain JIN4.

Characteristics	Results ^a^	Characteristics	Results ^a^
catalase reaction	+	citrate utilization	+
oxidase reaction	−	lecithin enzyme activity	+
starch hydrolysis	+	malonate utilization	−
MR test	−	cellulolytic activity	+
VP test	+	D-galactose utilization	−
gelatin liquefaction	+	D-arabinose utilization	−
nitrate reduction	+	D-mannose utilization	+
glucose fermentation	+	D-fructose utilization	+
H_2_S production	+	D-xylose utilization	+
produce IAA	+	ammonia production capacity	+
phosphorus-dissolving ability	+		

^a +^: Positive reaction; −: Negative reaction.

### 3.5 JIN4 has broad-spectrum inhibitory activity against pathogens

**Figure 6:**
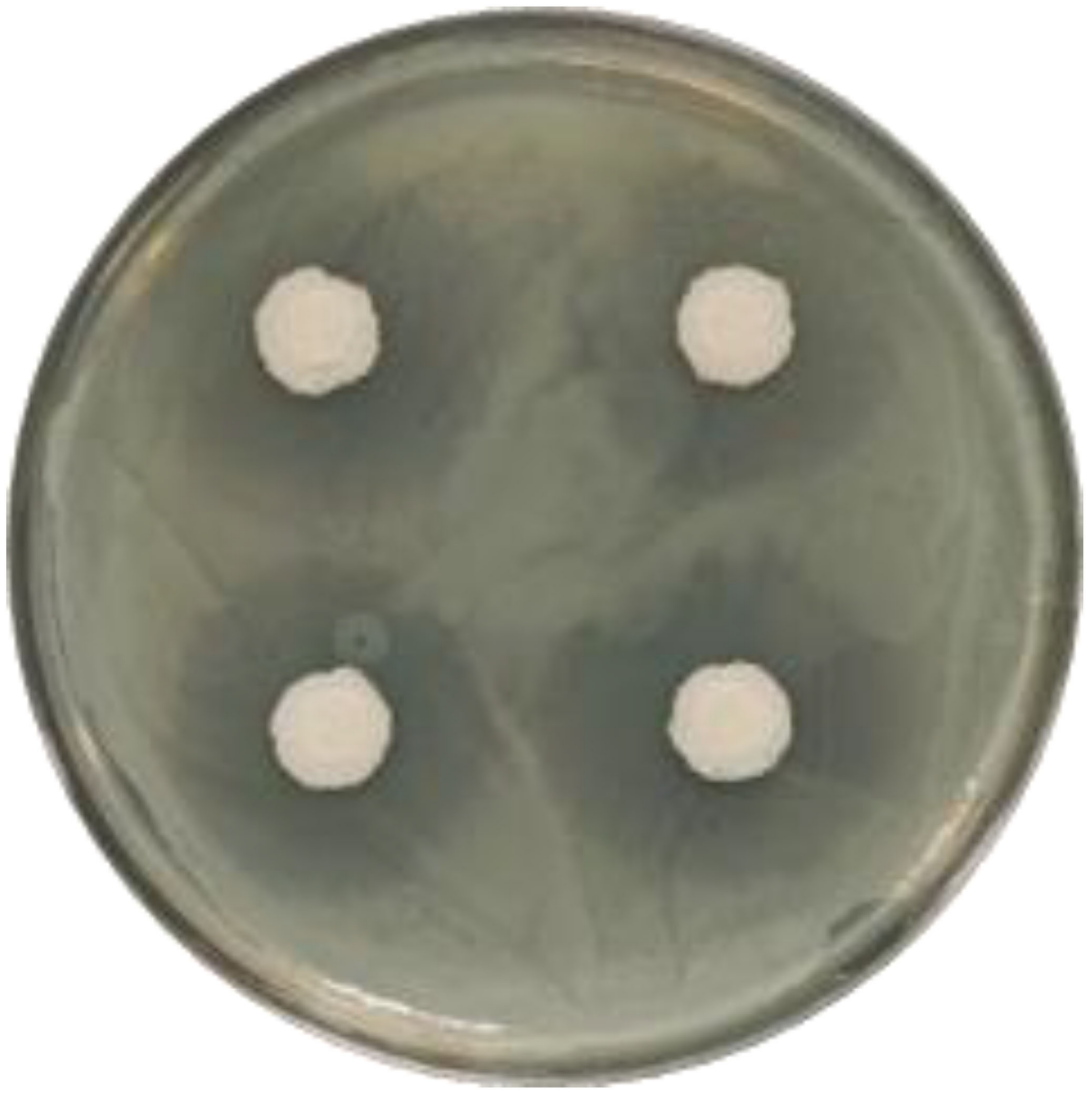
Zone of inhibition produced by bacteriophage-JIN4 that optimally antagonizes *Psa*.

The ability of strain JIN4 to inhibit eight different pathogens was measured to determine the antifungal range of strain JIN4. The results showed that strain JIN4 inhibited the pathogens (**Figure 6**). The strain showed different degrees of inhibition against various pathogens (**Table 4**), with strain JIN4 showing the highest antagonistic activity against pepper blight pathogen, with 89.32% inhibition. The inhibition of the cucumber wilt pathogen was weak, but the inhibition ratio was still over 65%.

**Table 4:** Relative efficacy of strain JIN4 against pathogenic fungi of common crops.

Fungi of common crops	Pathogen growth diameter/cm	Diameter after pathogen inhibition by JIN4/cm	Control efficiency/%
oil tea anthracnose pathogen	8.38 ± 0.76	1.55 ± 0.35	81.50
lotus root rot pathogen	8.30 ± 1.16	2.05 ± 0.47	75.30
cucumber wilt pathogen	8.87 ± 0.48	3.04 ± 1.24	65.73
rape botrytis pathogen	8.92 ± 0.34	1.31 ± 0.38	85.31
flax wilt pathogen	8.21 ± 0.59	1.92 ± 0.76	76.61
flax anthracnose pathogen	5.64 ± 1.21	1.49 ± 0.91	73.58
pepper blight pathogen	8.71 ± 0.86	0.93 ± 0.24	89.32
tobacco blight pathogen	8.67 ± 0.64	2.28 ± 0.73	73.70

### 3.6 Efficacy of JIN4 in controlling *Psa* C48 in petri dishes

**Figure 7:**
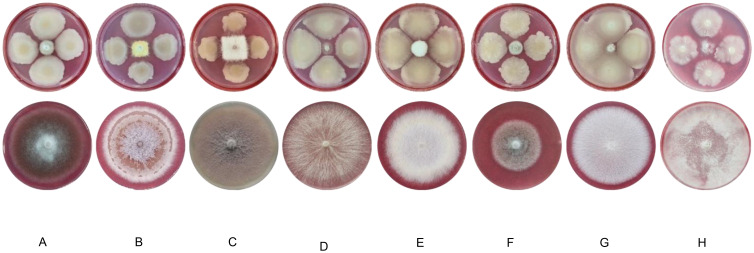
Fungal inhibition of common crop pathogenic fungi by strain JIN4. **(A)** oil tea anthracnose pathogen **(B)** lotus root rot pathogen **(C)** cucumber wilt pathogen **(D)** rape botrytis pathogen **(E)** flax wilt pathogen **(F)** flax anthracnose pathogen **(G)** pepper blight pathogen **(H)** tobacco blight pathogen.

A standoff culture of C48 was used to assess the inhibition capacity of the JIN4 in Petri dishes using the crossover method. The results showed that the outer diameter of the zone of inhibition of *Psa* C48 by JIN4 after 72 h of incubation on KB plates was 26.45 ± 1.47 mm, and the inner JIN4 growth diameter was 9.70 ± 0.29 mm, with a difference of 16.75 mm (**Figure 7**).

### 3.7 Efficacy of JIN4 under field conditions

After 14 days of treatment, the disease growth rates of the CK group, the JIN4 group, and the 3% benziothiazolino were 27.33%, 10.87%, and 7.93%, respectively. The efficacy of JIN4 was 60.22%, and the efficacy of 3% benziothiazolino was 70.99% (**Table 5**). JIN4 had a significant effect on the control of the kiwifruit bacterial canker. JIN4 is an endophytic bacterium isolated from kiwifruit in Hunan Province, China, which is better adapted to the environments of kiwifruit and Hunan Province. As an endophyte, JIN4 can effectively colonize kiwifruit plants, is not easily affected by the external environment, and forms a symbiotic relationship with the plants (Wicaksono et al., 2018; Ben Abdallah et al., 2016), which can allow symbiotic bacteria to play biological roles in the long term (Alsultan et al., 2019).

**Table 5:** Field efficacy of biocontrol bacteria JIN4.

Test groups	Pre-test disease index (%)	Post-test disease index (%)	Disease growth rate (%)	Control efficiency (%)
CK	26.56 ± 1.25	33.82 ± 1.63	27.33	–
JIN4	28.42 ± 1.26	31.51 ± 1.17	10.87	60.22 b
3% benziothiazolino	25.35 ± 1.60	27.36 ± 1.11	7.93	70.99 a

### 3.8 Effect of JIN4 on induced resistance in kiwifruit

**Figure 8:**
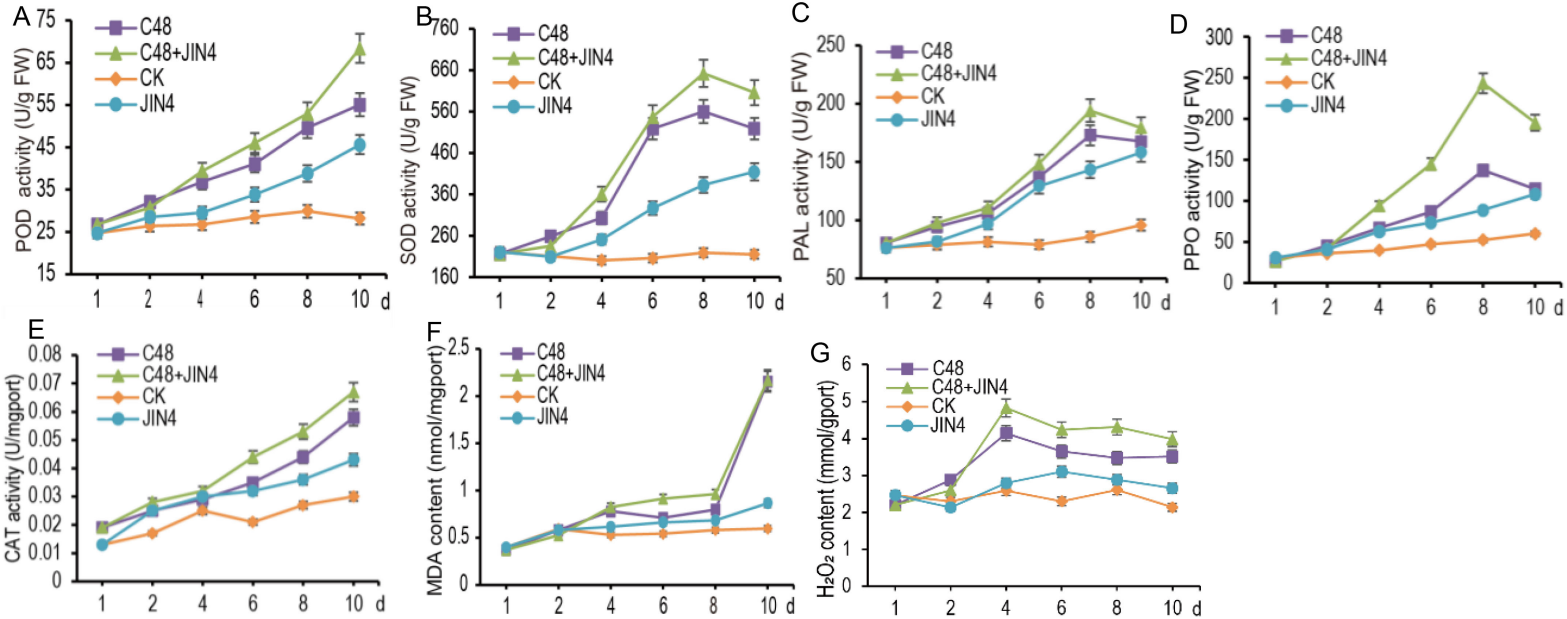
Response variables of kiwifruit seedlings under four different experimental conditions **(A)** POD, **(B)** SOD, **(C)** PAL, **(D)** PPO, **(E)** CAT activities, and **(F)** MDA, **(G)** H_2_O_2_ are compared.

The treatment group CK did not affect the five enzyme activities, and two metabolites of kiwifruit leaves, and the change trends were the same. The other three treatment groups rapidly increased the kiwifruit leaf enzyme activities, and the enzyme activities were increased in descending order by treatment groups C48+JIN4, C48, and JIN4. Both the C48+JIN4 and C48 treatment groups were able to rapidly increase the H_2_O_2_ content and MDA content of kiwifruit leaves (**Figure 8**). The results showed that JIN4 induced elevated activities of five antioxidant enzymes and elevated contents of two metabolites in kiwifruit, thereby increasing the resistance of kiwifruit plants to *Psa* infection.

## 4 Discussion

Antagonistic microorganisms have proven to be effective biological control agents in suppressing various plant diseases (Price-Christenson and Yannarell, 2023; Sunita, 2023; James et al., 2023; Abbas et al., 2024). In recent years, research on the use of biocontrol bacteria to prevent and control kiwifruit bacterial canker has been successful, and the colonization and control ability (Paparu et al., 2010; Zanon et al., 2024) of biocontrol bacteria is highly correlated with the sampling environment of the bacteria as well as the host (El-Deeb et al., 2013). The isolation of JIN4 in Hunan Province has resulted in a stronger adaptation to the local climate and soil environment, conferring an advantage in competing with local pathogens. Adaptation to the local microbial community structure and competitive pressure may also facilitate the occupation of an ecological niche in the kiwifruit inter-root or *in vivo*, thereby enhancing the inhibition of kiwifruit ulcer pathogens.

In the present study, *B. velezensis* JIN4 was isolated and its biocontrol potential was demonstrated. A comparison of the whole genome sequencing results of JIN4 with the CARD database revealed three resistance genes that potentially grant resistance to tetracycline, lincosamide, streptogramin antibiotics, etc. The resistance genes have the potential to enhance the survival of JIN4 in environments where antibiotics are applied, thereby increasing the effectiveness of biocontrol. They may also contribute to the reduction of the development of antibiotic resistance in pathogens and the management of the problem of pesticide resistance. Moreover, the joint action of JIN4 and antibiotics can facilitate disease control while reducing the necessity for chemical pesticides, which has a favorable impact on the pursuit of sustainable agriculture and environmental protection (Zhang et al., 2023). Additionally, the resistance genes of JIN4 offer a potential avenue for genetic engineering, which could facilitate the development of more efficacious biocontrol strains, thereby enhancing their utility in agricultural applications (Hemaiswarya and Doble, 2009; Jürgen et al., 2019).

Previous studies have shown that non-coding RNAs play a crucial role in the regulation of plant defense responses, especially when interacting with biocontrol factors. In biocontrol bacteria, ncRNAs can modulate plant immune responses and enhance plant resistance to bacterial pathogens by regulating the expression of disease resistance genes (Felden and Augagneur, 2021; Huang et al., 2023). In this study, we have predicted 80 ncRNAs, but their specific functions require further investigation. In addition, our study predicted a cluster of nine secondary metabolite synthesis genes of JIN4 was predicted to be a variety of peptidoglycan and polyketide glycosides and their precursors. Surfactin and fengycin can inhibit some fungi, bacteria, and viruses by acting on biofilms (Nair et al., 2016; Jin et al., 2018; Yadav et al., 2021). Butirosin is an aminoglycoside antibiotic effective against Gram-negative bacteria (Dion et al., 1972; Patrick and Patrice, 1986; Meng et al., 2022). Difficidin is a class of macrocyclic polyenolide phosphates with broad-spectrum antimicrobial activity that is thought to inhibit protein synthesis (Wu et al., 2015). Bacillaene inhibits bacteria and fungi by inhibiting protein synthesis (Patel et al., 2006; Berezhnaya et al., 2019; Pramod et al., 2021). Bacillibactin enhances the bioavailability of iron in the environment, promotes plant growth, and reduces pathogen abundance by competing for iron ions (Abergel et al., 2009; Lee et al., 2011; Nithyapriya et al., 2021). The cell walls of bacteria and fungi can be damaged by Baclysin (Wu et al., 2015; Ertekin et al., 2020). There are also two compounds, macrolactin H and mersacidin, whose functions are unknown. The identification of these nine secondary metabolite synthesis gene clusters suggests that JIN4 is capable of producing metabolites with antimicrobial activity. This will contribute to our comprehension of its mode of action in defense and control of pathogenic bacteria, and provide a target for further genetic and metabolic engineering in the future.

The inappropriate use of antibiotics has indeed fueled the rise of microbial drug resistance, leading to the emergence of multidrug-resistant bacteria (Ding et al., 2018). Various factors contribute to this concerning trend, including the excessive and indiscriminate use of antibiotics, inefficient diagnosis, and broad application in agricultural practices (Domingues et al., 2023). The biocontainment bacteria isolated from the location where the disease is endemic are more adaptable to the local climate, soil environment, microbial community structure and competitive pressure, etc. This enables them to compete more effectively with local pathogens, thus demonstrating superior control in agricultural production. JIN4 was tested not only for its ability to effectively colonize kiwifruit (Seifert et al., 1987; Kanu and Dakora, 2012; Mehmood et al., 2019; Hussain et al., 2015) but also for its ability to act as a bacterial fertilizer to promote plant growth (Hashem et al., 2019; Koua et al., 2020; Nithyapriya et al., 2021) and for its ability to improve plant disease resistance by enhancing leaf defense enzyme activities under *Psa* infection conditions after colonization. In the field test in this study, JIN4 still produced 60.22% inhibition of the bacterial canker of kiwifruit disease, significantly reducing the degree of ulcer disease caused by *Psa*. The discovery of strain JIN4 further confirms the potential application of *B. velezensis* to control kiwifruit bacterial canker, which is of great exploitation value in kiwifruit production.

It has been demonstrated that pathogenic bacteria possess conserved molecular structures, designated as pathogen-associated molecular patterns (PAMPs). Conversely, plants have evolved recognition molecules that can discern one or more PAMPs, which are classified as pattern recognition receptors (PRRs) (Triantafilou and Triantafilou, 2012). When pathogens invade plants, the plant’s PRRs recognize the pathogen’s PAMPs, inducing rapid defense responses such as an oxygen burst, salicylic acid signaling, and mitogen-activated protein kinase (MAPK) cascade responses (Zhang et al., 2007; Zipfel, 2009). It has been demonstrated that bacterial flagellin (flg22) can be recognized by Flgellin-sensitive 2 (FLS2) in Arabidopsis thaliana. This recognition initiates endocytosis, which transmits the signal to the interior of the cell. Subsequently, the downstream transcription factor WRKY is activated through the MAPK cascade reaction. This pathway is designated PAMP-triggered immunity (PTI), which plays a pivotal role in the defense response of plants (Tena et al., 2001). However, plants subjected to adverse conditions will produce excessive ROS, which will have a deleterious impact on their normal physiological activities (Schneide et al., 2019). A protective enzyme system is present in plants, which is capable of removing ROS. This has the effect of alleviating damage caused by excessive ROS and enhancing the resilience of plants to adversity. The protective enzyme system comprises SOD, CAT and POD, which are capable of removing H_2_O_2_, O_2_^-^, and other reactive oxygen species. The key enzymes involved in the synthesis of secondary plant materials are PPO and PAL (Dvorak et al., 2021). These have significant functions in the growth and development of the plant, as well as in the plant’s ability to resist external threats. The enzymes are also involved in the plant’s defense against pests and diseases, as well as in its capacity to withstand stress. An increase in the level of MDA is indicative of damage to the cell membrane, which serves as a key indicator of the extent of damage to the plant. H_2_O_2_ is a vital signaling molecule within the plant body, playing a crucial role in the plant’s defense response. Studies have shown that the use of induced plant resistance in the production of kiwi fruit is also an effective way of reducing the damage caused by canker (Jong et al., 2019; Reglinski et al., 2022). In addition, these results showed that JIN4 promoted the generation of induced resistance in kiwifruit *in vivo* with progressively higher CAT, PPO and SOD activities, and that JIN4 showed better induced efficacy in the presence of *Psa*, with a more intense process of enzyme synthesis and accumulation of secondary metabolites, providing a rationale for the treatment of kiwifruit canker disease by JIN4.

## 5 Conclusion

This study represents the first instance in which antibiotic resistance genes and secondary metabolite synthesis gene clusters have been excavated from the *B. velezensis* genome, with the objective of verifying its stability and adaptability for effective control of *Psa*. The pathway of action and efficacy of biocontrol, as well as the evaluation of biocontrol effects, must be clarified as prerequisites for the development of biocontrol products. JIN4 produces metabolites against pathogenic bacteria and induces antioxidant enzyme activities that enhance plant resistance to pathogenic bacteria. In conclusion, the JIN4 strain has been demonstrated to exert a beneficial influence on the control of kiwifruit ulcer disease, and it is therefore a promising candidate for further development and application in kiwifruit production.

## Data Availability

The original contributions presented in the study are publicly available. This data can be found here: https://www.ncbi.nlm.nih.gov/genbank/, accession numbers CP087137.1 and MZ336040.1.
