# Supplementary material for: Isolation and identification of a novel Bacillus velezensis strain JIN4 and its potential for biocontrol of kiwifruit bacterial canker caused by Pseudomonas syringae pv. actinidiae
Source: Front Plant Sci. 2024 Dec 23;15:1513438. doi: 10.3389/fpls.2024.1513438 (PMC11701161; doi:10.3389/fpls.2024.1513438)
Supplement: Supplementary file 1 [file DataSheet1.docx]

Supplementary Material

# Supplementary Tables and Figures

## Supplementary Tables

**Supplementary Table 1.** Screening strain JIN4.

| Isolation Strain | Inner diameter of Zone of inhibition (mm) | Outer diameter of zone of inhibition (mm) | Diameter difference of Zone of inhibition (mm) |
| --- | --- | --- | --- |
| JIN1 | 14.94±2.10 | 17.19±1.40 | 2.25 |
| JIN2 | 11.75±0.69 | 25.85±0.73 | 14.10 |
| JIN3 | 10.97±0.87 | 12.32±0.53 | 1.35 |
| JIN4 | 9.70±0.29 | 26.45±1.47 | 16.75 |
| JIN5 | 14.25±0.79 | 17.68±1.23 | 3.43 |
| JIN6 | 12.24±0.72 | 18.21±1.87 | 5.97 |

## Supplementary Table 2. Non-coding RNA prediction.

| RNA types | | Amount | Average length /bp | Total length /bp | Percentage of genome /% |
| --- | --- | --- | --- | --- | --- |
| rRNA | 5S rRNA | 9 | 116 | 1044 | 0.0267 |
|  | 16S rRNA | 9 | 1550 | 13950 | 0.3573 |
|  | 23S rRNA | 9 | 2930 | 26370 | 0.6754 |
| tRNA | | 86 | 77.1 | 6633 | 0.1699 |
| other ncRNA | | 80 | 142.5 | 11403 | 0.2920 |

**Supplementary Table** **3.** Statistics of CRISPR prediction results for strain JIN4.

| CRISPR id | Start | End | Repeat sequence copy number | Average length of repeated sequences /bp | Number of interval sequences | Average length of interval sequences /bp |
| --- | --- | --- | --- | --- | --- | --- |
| CRISPR.1 | 458893 | 459152 | 5 | 20 | 4 | 40 |
| CRISPR.2 | 729388 | 731919 | 39 | 21 | 38 | 45 |
| CRISPR.3 | 734914 | 735639 | 10 | 33 | 9 | 44 |
| CRISPR.4 | 736528 | 738284 | 23 | 20 | 22 | 58 |
| CRISPR.5 | 1174213 | 1174277 | 2 | 23 | 1 | 19 |
| CRISPR.6 | 1218721 | 1219478 | 10 | 29 | 9 | 52 |
| CRISPR.7 | 1263786 | 1264024 | 5 | 23 | 4 | 31 |
| CRISPR.8 | 2649039 | 2649344 | 8 | 20 | 7 | 20 |
| CRISPR.9 | 2812319 | 2812455 | 4 | 20 | 3 | 19 |
| CRISPR.10 | 3282512 | 3282577 | 2 | 19 | 1 | 28 |
| CRISPR.11 | 3445695 | 3445792 | 3 | 20 | 2 | 19 |
| CRISPR.12 | 3445702 | 3445956 | 4 | 27 | 3 | 49 |

**Supplementary Table 4.** Combined antibiotic resistance.

| Gene id | ARO accession | Resistance |
| --- | --- | --- |
| GE001168 | ARO:3000178 Tet(K) | tetracycline antibiotic |
| GE000505 | ARO:3002814 clbA | lincosamide antibiotic, pleuromutilin antibiotic, macrolide antibiotic, oxazolidinone antibiotic, streptogramin antibiotic, and phenicol antibiotic |
| GE000525 | ARO:3004476 vmlR | lincomycin; streptogramin A virginiamycin |

## Supplementary Figures


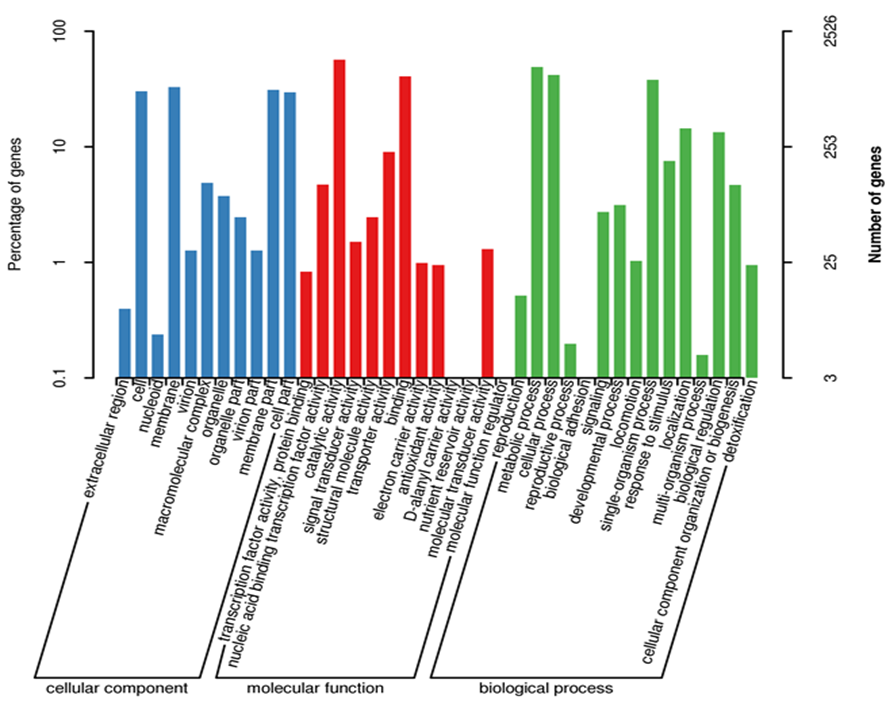


**Supplementary Figure 1.** GO function annotated classification statistics chart.

Note: The horizontal axis indicates the content of each classification of GO analysis, the primary vertical axis on the left is the percentage of the number of genes, and the secondary vertical axis on the right is the number of genes. This figure shows the enrichment of each secondary function gene in GO analysis in all gene backgrounds, which can reflect the status of each secondary function.


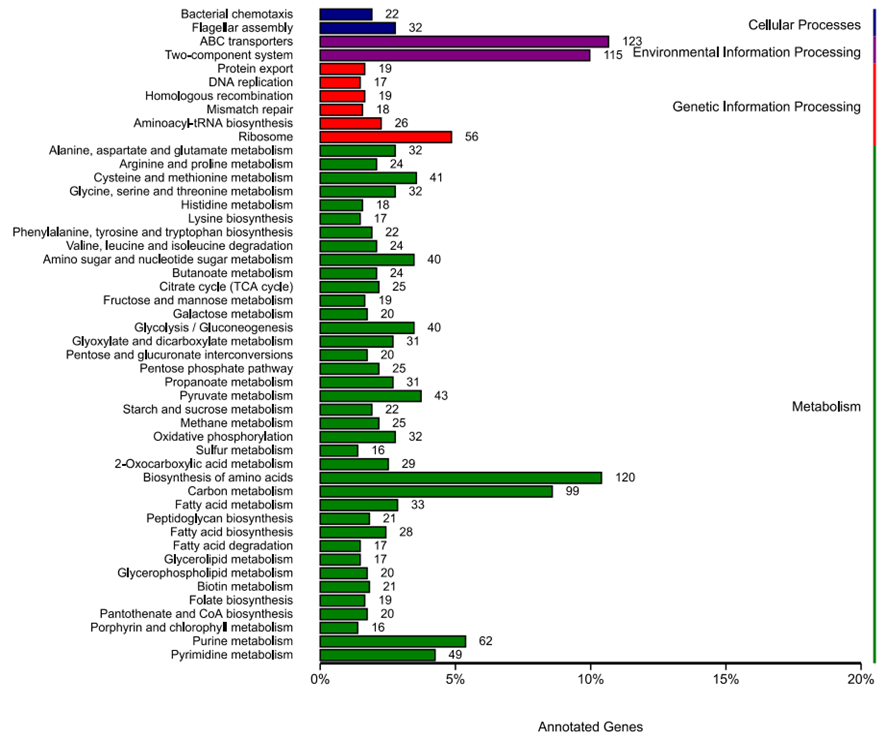


**Supplementary Figure 2.** Statistical chart of KEGG functional annotation classification.

Note: The vertical axis indicates the results of KEGG analysis of secondary classification, and the horizontal coordinate is the percentage of the percentage.
